# Atopy and Elevation of IgE, IgG3, and IgG4 May Be Risk Factors for Post COVID-19 Condition in Children and Adolescents

**DOI:** 10.3390/children10101598

**Published:** 2023-09-25

**Authors:** Robert Walter Körner, Ole Yannick Bansemir, Rosa Franke, Julius Sturm, Hormos Salimi Dafsari

**Affiliations:** 1 Department of Pediatrics, Faculty of Medicine and University Hospital Cologne, University of Cologne, 50937 Cologne, Germany; 2 Center for Rare Diseases, Faculty of Medicine and University Hospital Cologne, University of Cologne, 50937 Cologne, Germany; 3 Max-Planck-Institute for Biology of Ageing, 50931 Cologne, Germany; 4 Cologne Excellence Cluster on Cellular Stress Responses in Aging Associated Diseases (CECAD), University of Cologne, 50931 Cologne, Germany

**Keywords:** post COVID-19 condition, atopic disease, long COVID, children, adolescents, SARS-CoV-2

## Abstract

SARS-CoV-2 infection causes transient cardiorespiratory and neurological disorders, and severe acute illness is rare among children. Post COVID-19 condition (PCC) may cause profound, persistent phenotypes with increasing prevalence. Its manifestation and risk factors remain elusive. In this monocentric study, we hypothesized that atopy, the tendency to produce an exaggerated immunoglobulin E (IgE) immune response, is a risk factor for the manifestation of pediatric PCC. We present a patient cohort (n = 28) from an early pandemic period (2021–2022) with comprehensive evaluations of phenotypes, pulmonary function, and molecular investigations. PCC predominantly affected adolescents and presented with fatigue, dyspnea, and post-exertional malaise. Sensitizations to aeroallergens were found in 93% of cases. We observed elevated IgE levels (mean 174.2 kU/L, reference < 100 kU/L) regardless of disease severity. Concurrent Myalgic Encephalomyelitis/Chronic Fatigue Syndrome (ME/CFS) was found in 29% of patients that also faced challenges in school attendance. ME/CFS manifestation was significantly associated with elevated immunoglobulin G subclasses IgG3 (*p* < 0.05) and IgG4 (*p* < 0.05). A total of 57% of patients showed self-limiting disease courses with mean recovery at 12.7 months (range 5–25 months), 29% at 19.2 months (range 12–30 months), and the rest demonstrated overall improvement. These findings offer additional insights into immune dysregulation as a risk factor for pediatric PCC.

## 1. Introduction

SARS-CoV-2 infection causes an acute illness with mostly upper respiratory and chest infections, anosmia, flu-like fatigue, as well as muscle and joint pains [1]. At the onset of the COVID-19 pandemic in 2020, children and adolescents were observed to face a lower risk of transmission of SARS-CoV-2 in schools, leading to the assumption that they may be less affected by COVID-19 compared to middle-aged adults [2]. However, as the pandemic progressed, it became evident that (i) children and adolescents may develop severe acute health issues even at lower transmission risk, and (ii) individuals at any age may have lasting symptoms arising from an acute infection. These issues included conditions such as MIS-C (Multisystem Inflammatory Syndrome in Children) and secondary effects resulting from lockdown restrictions, home schooling, and mental health [3]. In parallel to adults, post COVID-19 condition (PCC) emerged as a significant disease entity, characterized by a prolonged period of symptoms that may profoundly impact daily activities.

PCC is defined by a history of confirmed SARS-CoV-2 infection, with one or more persisting physical symptom(s) for a minimum duration of 12 weeks after initial testing that do not arise from an alternative diagnosis. These symptoms impact everyday functioning of the central nervous system that include concentration deficits (‘brain fog’), headaches, perception disorders, cognitive impairments, speech disorders, or anxiety. Additional peripheral or autonomous nervous system disorders include recurrent fever, body temperature instability, postural tachycardia, and gastrointestinal symptoms, that may fluctuate or relapse over time [4]. To what extent sudden exhausting events may trigger any such relapse of fatigue remains elusive.

While reports of PCC in children and adolescents began to surface over time, prevalence of PCC in children and adolescents exhibit considerable variability, with a predominant impact on adolescents [5]. A comprehensive study conducted in the United Kingdom revealed a prevalence of 4.4% among children aged 5–11 years and 13.3% among those aged 12–17 years [6]. A study conducted in Germany reported an overall prevalence of 1.7% among children and adolescents under 18 years, similarly highlighting a higher risk of PCC development in the age group of 13–17 years [7]. 

Recurring hypotheses for the pathophysiology of PCC include tissue damage during acute COVID-19 illness as well as persistent, pathological inflammation, e.g., due to viral persistence in tissues, immune dysregulation, and autoimmunity [8,9]. Recent reports have shown that PCC can affect individuals at any age with a particular rise in middle-aged patients which suggested acquired immune dysregulation throughout life [10,11]. Any onset during childhood in PCC cases may raise questions to a genetic susceptibility towards immune dysregulation. The most common factor in childhood-onset immune dysregulation is atopy, the inherited tendency to produce specific immunoglobulin E (IgE) antibodies in response to common environmental factors, which is associated with a progressive course in obtaining allergic diseases such as rhinitis, asthma, or eczema [12]. Other reasons for childhood-onset immune dysregulation may include ultra-rare genetic diseases that perturb innate immunity pathways [13,14]. While atopic disease may not be a risk factor for developing acute COVID-19 illness [15], a questionnaire-based survey from the Italian Pediatric Society for Allergy and Immunology (SIAIP) has indeed previously found less than 20% PCC in patients under the treatment of mostly primary pediatricians in Italy [16]. The pathomechanisms in the manifestation of pediatric PCC and its risk factors remain elusive.

Here, we report a monocentric study on 28 patients with suspicion of PCC that were recruited at an early time period of the COVID-19 pandemic and revisited throughout the pandemic in a cohort of young individuals from the greater region of Cologne, Germany. 

The primary hypothesis in this project was that deep phenotyping and molecular investigations of organ function in pediatric PCC patients reveal signs of tissue damages that may have manifested at either acute COVID-19 illness or during the course of PCC. We aimed to include detailed phenotypic findings based on a modified version of the International Consensus Criteria [17], molecular investigations from peripheral blood, and pulmonary function tests in patients with PCC who presented to our outpatient clinic, in order to reveal specific abnormalities and potential patterns for particularly severe disease courses with ME/CFS in our pediatric cohort.

The secondary hypothesis was that atopy is a risk factor for the manifestation of PCC. For this analysis, we aimed to conduct immunological studies with immunoglobulin levels and lymphocyte subcounts, and performed subgroup analyses to investigate associations of immunological abnormalities with manifestation of ME/CFS as particularly severe disease courses of PCC.

As pediatric PCC is a rare disorder with severe impact on activities of daily living, these findings may provide groundwork for the counseling of patients and families and future studies in the field. 

## 2. Materials and Methods

### 2.1. Study Design

We recruited children and adolescents from our dedicated PCC out-patient clinic during an early time period during the COVID-19 pandemic, beginning from August 2021 until July 2022. The patients were revisited for at least two appointments throughout March 2023.

The study was approved by the Institutional Review Board of the Medical Faculty at the University of Cologne, Germany (protocol code 23-1077; protocol code 20-1711). All patients and/or guardians have given consent.

The inclusion criteria were (i) suspicion of PCC following an acute infection with COVID-19 based on patient history, (ii) confirmed COVID-19 infection through a polymerase chain reaction (PCR) test, (iii) patients below 18 years of life at initial presentation, and (iv) patients with at least two follow-up appointment during the time-frame mentioned above. Patients were excluded from the analysis who had not received previous PCR testing or were found with an alternative diagnosis. 

### 2.2. Data Extraction and Event Variables

The data were extracted from patients’ digital health records and entered in a completely anonymized manner. The collection of parameters during diagnostic procedures were consistently standardized across all cases. The dataset included demographic information such as age and gender, as well as details on acute COVID-19 characteristics. PCC onset and progression were documented by specialist physicians and families with a modified version of the International Consensus Criteria for ME/CFS (Appendix A Table A1) [17]. Additional data included pulmonary function tests, and laboratory investigations including clinical chemistry, markers of cardiac function, thyroid hormones, inflammation parameters, and coagulation studies. Immunological studies encompassed immunoglobulin levels, allergen-specific IgE levels, IgG to SARS-CoV-2 Spike RBD, complete blood count, and the analysis of lymphocyte subsets.

### 2.3. Data Analysis

The statistical analysis was conducted with Prism for macOS (version 9, GraphPad Software, LLC, Boston, MA, USA) and SPSS for MacOS (version 28, IBM Corp., Armonk, NY, USA). The group comparisons were performed using the Mann–Whitney test and ANOVA (*p* < 0.05). The correlation analyses were performed using Pearson’s coefficients (two-tailed test of significance).

## 3. Results

### 3.1. Patient Cohort and Disease Characteristics

A cohort of 70 patients, who had experienced acute illness during the early pandemic period, initially presented to our out-patient clinic between August 2021 and July 2022. These patients were referred by regional primary care pediatricians. As our primary hypothesis followed a clear temporal link to a previous acute COVID-19 illness, this led to an exclusion of patients without PCR testing during acute illness. We focused our analyses on the remaining 28 recruited patients with suspicion of PCC and clear link to COVID-19 based on PCR testing. In this group, we here report patients with at least two follow-up appointments to evaluate disease courses.

**Figure 1:**
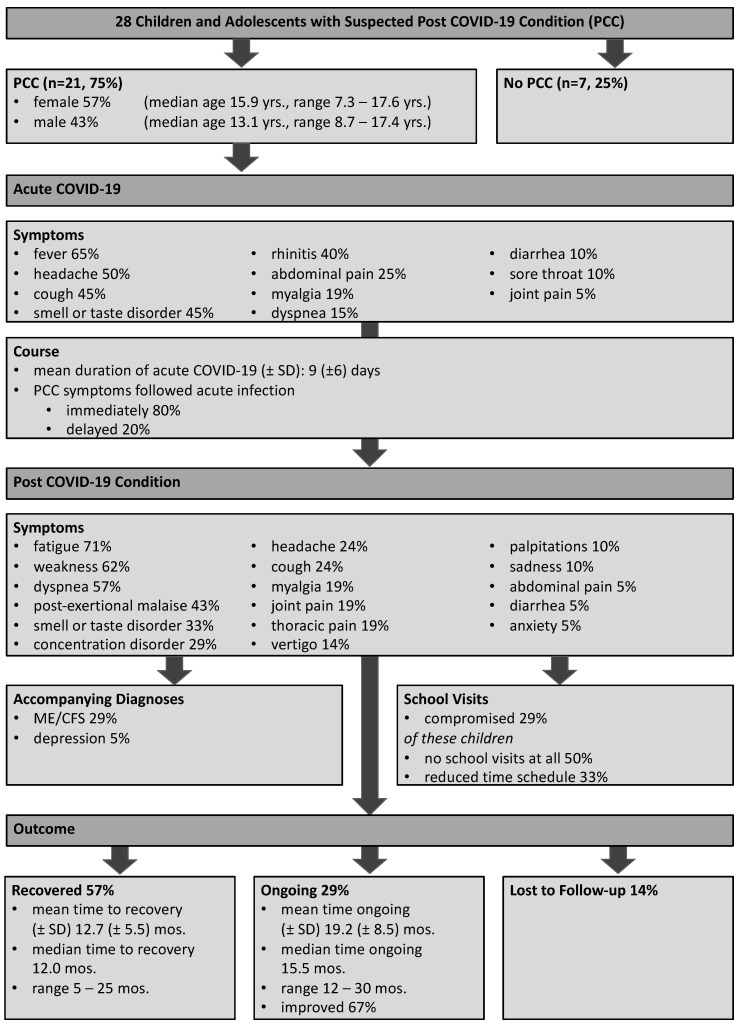
Overview on the presentation of 28 children and adolescents with suspected PCC and the results from the algorithm at our dedicated specialist clinic regarding presentation of PCC phenotypes, accompanying diagnoses, school visits, and outcome.

From a total of 28 patients with full inclusion criteria, 21 patients (75%) were diagnosed with PCC (Figure 1). There was a slight female predominance in our cohort (57% females, 43% males), and the majority of patients were adolescents older than 10 years of age (n = 16, 76%). Median age was 13.7 years (range 7.3–17.6 years). Chronic diseases were observed in 52% of the patients (n = 11), including allergic diseases (n = 5, 24%), respiratory diseases (n = 4, 19%), cardiac diseases (n = 3, 14%), mental diseases (n = 3, 14%, including anxiety in two cases and chronic leg pain in one case), nephrological diseases (n = 1, 5%), and endocrine diseases (n = 1, 5%). 

From a total of 28 included patients, 7 patients were evaluated with an alternative underlying reason that was unrelated to SARS-CoV-2 infection. Differential diagnoses included bronchial asthma, humoral immune deficiency, psychiatric disorders, and periodic fever syndrome. These cases were excluded from further analysis.

### 3.2. Acute COVID-19 Disease Course

A previous infection with SARS-CoV-2 had been confirmed by PCR testing in every case. The most common symptoms during acute COVID-19 were fever (n = 14, 65%), headache (n = 11, 52%), cough (n = 9, 43%), and impaired smell or taste (n = 9, 43%). The severity of the acute disease was graded by the patients as follows: mild in 29% (n = 6), moderate in 57% (n = 12), and severe in 14% (n = 3) of the patients. None of the patients were hospitalized during acute illness. The majority of patients (n = 15, 71%) remained bedridden during acute illness. The mean duration of acute symptoms was 9 days (SD ± 6 days).

### 3.3. Post COVID-19 Condition Disease Course

In our cohort of 21 patients, there was an average duration of 192 days from onset of acute COVID-19 illness until inclusion into our study (minimum 76 days, maximum 356 days). In patients with initial durations from illness to inclusion below 90 days, we diagnosed PCC on follow-up appointments after 90 days. In 80% of the cases (n = 17), symptoms of PCC developed seamlessly after the acute COVID-19 disease. A total of 20% of patients (n = 4) reported a full recovery between acute illness and PCC, ranging from a few days up to six weeks. The most common symptoms included flu-like fatigue (n = 15, 71%), weakness (n = 13, 62%), dyspnea (n = 12, 57%), and post-exertional malaise (n = 9, 43%). One-third of the patients met the international consensus criteria for ME/CFS [8]. One patient was diagnosed with depression. Loss of weight or appetite were not reported in any of our patients. Physical examination at clinical presentation during our dedicated specialized clinic for pediatric PCC included thorough cardiorespiratory and neurological examinations and did not reveal any relevant pathologic findings such as signs of pulmonary disease, focal neurological deficits, or heart murmurs.

The compromised health condition prevented 28% patients (n = 6) from attending school regularly. Among these children, three patients had no school attendance at all, two patients attended school on a reduced schedule ranging from one to four attendance days per week, and one patient reported five days per week but with around one to two school hours missed per day.

A total of 57% of the patients (n = 12) had experienced a recovery with an average duration of 12.7 months (range 5–25 months). The majority of these patients reported a rather gradual improvement in this self-limiting course, although some still experienced occasional concentration deficits even after recovery. A second group comprising 29% (n = 6) demonstrated a continuing course of PCC with an average duration of 19.2 months (range 12–30 months) at the time of analysis. Among those, 67% (n = 4) reported a significant improvement in their symptoms. The rest of the patients (n = 3, 14%) dropped out of follow-up investigations after two appointments.

### 3.4. Pulmonary Function Tests for Dyspnea

To investigate for abnormalities of pulmonary function related to reported dyspnea in patients, we conducted spirometry which showed no cases of restrictive or obstructive ventilatory disorders (mean ± SD: FVC 96.3% ± 11.6, FEV1 96.4% ± 10.6, FEV1/FVC 100.2% ± 8.6). Bronchodilator reversibility testing was negative in all cases. FEV1 and FVC were not diminished in subgroups based on the presence of dyspnea, cough, thoracic pain, weakness, fatigue, post-exertional malaise, and ME/CFS. Oxygen saturation was normal in all patients. Fractional exhaled nitric oxide (FeNO) was elevated in three patients (≥20 ppb). One of these patients reported dyspnea and none reported coughing.

### 3.5. Molecular Investigations for Organ Dysfunction

Table 1 illustrates an overview of the molecular investigations for organ dysfunction from peripheral blood. These standard analyses included electrolytes (sodium, potassium, chloride, calcium, phosphate), liver function parameters (alanine aminotransferase, aspartate aminotransferase, γ-glutamyltransferase, glutamate dehydrogenase, cholinesterase, bilirubin), skeletal and cardiac muscle parameters (creatine kinase, creatine kinase-MB, troponin T-high sensitive, NT-proBNP), kidney function parameters (total protein, albumin, creatinine, blood urea nitrogen, uric acid), and others (blood sugar, lactate dehydrogenase, iron, ferritin, transferrin, transferrin saturation, and lipase). These molecular investigations revealed no abnormalities in organ functions.

Thyroid-stimulating hormone and free thyroxine T4 levels indicated normal thyroid function for all cases. Coagulation studies, including international normalized ratio, activated partial thromboplastin time, fibrinogen, and D-dimer, were normal in all patients. There were no signs of a systemic inflammation as indicated by C-reactive protein or erythrocyte sedimentation rate in any cases.

### 3.6. Immunological Diagnostics for Immune Dysregulation

We did not observe any alterations in the levels of immunoglobulins G, A, and M. Testing for immunoglobulin G subclasses (G1-G4) also showed no alterations. IgG against SARS-CoV-2 Spike RBD was detected in all but two individuals, displaying significant variability (mean 1233 BAU/mL, SD ± 1707 BAU/mL, median 326 BAU/mL, range 0–5619 BAU/mL). Elevated IgE levels exceeding 100 kU/L were found in 43% of the patients. Sensitizations to aeroallergens were prevalent in 93% of the cases, with the most common sensitizations observed towards dogs (87%), horses (60%), cats (46%), and the house dust mite *D. farinae* (40%). No sensitizations against fungi were detected.

The complete blood count revealed no abnormalities in leukocytes, thrombocytes, erythrocytes, and erythrocyte parameters, including hematocrit, hemoglobin, red cell distribution width, mean corpuscular volume (MCV), mean corpuscular hemoglobin (MCH), and mean corpuscular hemoglobin concentration (MCHC), across all cases. However, in one patient, there was a slight decrease in neutrophil count, while the monocyte count was slightly elevated in four patients. Additionally, one patient exhibited an elevation in the eosinophil count, and two patients in the lymphocyte count. Lymphocyte subset analysis showed no remarkable findings except for an increased level of natural killer cells in one patient (Table 2).

As our secondary hypothesis was that atopy is a risk factor for PCC manifestation, we analyzed immunoglobulin E (IgE) levels in our cohort and indeed observed elevated IgE levels at 174.2 kU/L (reference range < 100 kU/L). To first test if this finding of IgE elevation was driven by a few patients with allergic diseases, we analyzed the five patients with reported allergic diseases (average IgE levels of 242.12 kU/L) and non-allergic patients (average IgE levels of 148.13 kU/L). This finding shows elevation IgE levels regardless of reports of allergic diseases. Subgroup analyses did not reveal a significant association of reported allergic disease and IgE levels (*p* = 0.398). Next, we tested for the hypothesis that IgE elevation was a biomarker for disease severity. There was no significant correlation between IgE elevation and severe disease courses with ME/CFS (r = −0.234, *p* = 0.351). This led us to the finding that IgE elevation is a general phenomenon in our cohort of PCC patients regardless of disease severity.

Next, we analyzed immunoglobulin G levels including subclasses IgG1–IgG4. There were no elevated IgG levels on average in our cohort (IgG 10.66 g/L, IgG1 6014 mg/L, IgG2 2865 mg/L, IgG3 863 mg/L, IgG4 538 mg/L). The five patients with ME/CFS showed average IgG3 levels at 1835 mg/L and average IgG4 levels at 881 mg/L, both of which can be considered as mild elevations, while the rest of the patients had IgG3 levels at 538 mg/L and IgG4 levels at 424 mg/L. Subgroup analyses did indeed reveal that severe disease courses with ME/CFS were significantly associated with increases in IgG3 levels (*p* = 0.022) and IgG4 levels (*p* = 0.047). 

## 4. Discussion

### 4.1. General Cohort Characteristics in Pediatric and Adolescent PCC

This monocentric study on pediatric PCC patients presents a cohort from an early time period of the pandemic that included predominantly individuals in adolescence rather than childhood which aligns with findings from previous studies [6,7]. While the collective of patients with PCC in our dedicated clinic is far larger than the reported 28 patients, we only included patients (i) from an early period of the pandemic, (ii) after confirmed acute COVID-19 illness based on positive PCR testing, and (iii) with repeated presentation to evaluate disease course and recovery. These inclusion criteria were designed to incorporate a clear link to PCC based on patient history and testing to address our primary hypothesis investigating organ dysfunction resulting from acute COVID-19 illness. Throughout the pandemic PCR tests for acute illness were performed based on capacities of hospitals, schools, or private companies. By the end of 2022, PCR testing in Germany was largely reduced to a minimum of cases with severe hospitalization [18]. We discuss that while setting the inclusion criteria to positive PCR testing clearly limits the number of patients in an already limited collective of children, it still serves as a clear link of PCC to acute COVID-19 illness to investigate any organ dysfunctions or immune dysregulation that is the purpose of this study.

In most cases, PCC symptoms manifested immediately after acute COVID-19 disease, and only few instances appeared with brief delay between acute illness and beginning of PCC symptoms. The underlying reason for this variability in our cohort and the pathophysiology of PCC remains elusive and is currently discussed to differ from the acute inflammatory process of COVID-19 [19]. Potential factors contributing to the development of PCC include viral persistence, dysfunctional autonomous neuronal signaling, vasculopathies, coagulation abnormalities, microbiota dysbiosis, and chronic inflammatory processes involving immune dysregulation and autoimmunity [19,20]. However, the duration from acute COVID-19 illness until recruitment was at an average of 192 days, potentially indicating the inappropriate delay of diagnoses, despite manifesting the symptoms of PCC mostly immediately following acute COVID-19 illness.

We did not identify organ dysfunctions from molecular investigations in peripheral blood that may arise from tissue damage during acute COVID-19 illness in our patient collective. The reported symptoms of acute COVID-19 illness in our patients who later developed PCC were similar to the known symptoms of acute COVID-19 as previously published [21]. A comprehensive meta-analysis indicated that severe initial symptoms during acute infections may serve as a risk factor for PCC in children, similar to findings observed in adults [22]. The meta-analysis also identified older age, female gender, and poor mental or physical status as additional risk factors for PCC. Interestingly, our small cohort also reflected an increase in age (adolescence) and female gender as a risk factor for PCC. Notably, approximately half of the patients in our cohort had pre-existing chronic diseases, with respiratory conditions being the most commonly reported. Comorbidities were identified as independent risk factors for PCC in a larger pediatric cohort, corroborating our findings [23]. 

### 4.2. Neuropsychological Phenotypes

The most prevalent symptoms included fatigue, weakness, dyspnea, and post-exertional malaise, which are often considered as primary indicators of the condition [22]. The three patients with mental disease before PCC manifestation from a total of 21 patients might grant insight into concurrent neuropsychological disorders in pediatric PCC. While the additional phenotypes with PCC differed significantly from earlier neuropsychological disease, we discuss that any concurrent neuropsychological disorder may have an additional emotional impact on children and families.

The presented PCC cohort indicates the concomitant manifestation of ME/CFS as a particularly severe disease course with school absenteeism. The presence of ME/CFS represents one of the most challenging aspects of PCC, as it significantly limits daily activities over an extended period and can indeed lead to a secondary dysthymia or depression especially at adolescence. Several environmental triggers have been discussed to aggravate courses, for instance, puberty crises, parental stress, or loss of social connections [24]. We report frequent school absenteeism among patients with PCC-associated ME/CFS. While this has indeed been discussed before, there are few real-life data yet on the true extent on ME/ECFS and our data may serve as groundwork for future studies.

Parents and patients commonly expressed difficulties in finding doctors familiar with ME/CFS, and their complaints were often dismissed or considered invalid, further exacerbating the strain on patients. ME/CFS has long been recognized to occur in conjunction with post-viral syndromes and was discussed as involving dysregulation of the immune system, metabolism, and the autonomic nervous system [25]. The emergence of specialized centers such as ours is relatively sparse, and we argue that any resolute support of children and adolescents with PCC requires composition and supplement of specialized pediatric PCC centers. 

### 4.3. Discrepancy in Self-Reported Dyspnea and Unremarkable Pulmonary Function

Self-reported dyspnea was reported frequently as a symptom of PCC in our patient cohort (n = 12, 57%). We conducted pulmonary function test to investigate for the presence of abnormal lung ventilation. While pulmonary function was normal in all patients, we discuss that this finding does not necessarily rule out the presence of lung pathologies. A separate study utilizing low-field-strength MRI revealed persistent pulmonary dysfunction, characterized by a lower proportion of ventilated and perfused lung parenchyma, in children, adolescents, and adults with PCC and also those who had recovered from COVID-19 [26]. 

Furthermore, in adults, dual-energy computed tomography (CT) identified widespread microangiopathy in the majority of PCC patients following COVID-19 hospitalization [27]. Additionally, another study in adults demonstrated persistent neutrophil inflammation with increased peripheral total neutrophil numbers and interstitial lung changes at 3–6 months after COVID-19 recovery [28]. In contrast, children with dyspnea in our cohort did not exhibit signs of neutrophilic inflammation. We also measured FeNO (fractional exhaled nitric oxide) levels, which were elevated in only three cases and did not warrant further investigations. Notably, FeNO has not proven to be a useful biomarker in the immediate follow-up of adult COVID-19 patients [29]. 

Adult PCC patients with little to no dyspnea have previously been reported with ventilation defects in ^129^Xe MRI that correlated with lower six-minutes walking distance and oxygen saturation measurements after exertion. However, these findings did not correlate with disease-specific testing for dyspnea, including the St George’s Respiratory Questionnaire (SGRQ) or modified MRC dyspnea scale [30]. Another study reported an improvement in ventilation disorder from ^129^Xe MRI findings and SGRQ test from 7 to 14 months after infection [31]. While there are no reports yet for ^129^Xe MRI in children with PCC, we discuss that such investigations in children would indeed grant insight into the developing lung during PCC in future studies.

An additional hypothesis in the field has revolved around disorders of the autonomic nervous system as a factor for dyspnea. While we have not investigated potential markers for small fiber neuropathy, we do discuss that future studies are required to find definitive diagnostic tests to detect and monitor lung diseases in children following COVID-19.

### 4.4. Molecular Investigations

As presentation with suspected diagnosis of PCC requires a comprehensive assessment to rule out differential diagnoses, our goal was to indeed analyze for any significant deviations in standard laboratory blood investigations. A particular emphasis lied on the interpretation of age- and gender-appropriate laboratory results, and cases with deviations from standard ranges should not be necessarily excluded by definition. In this study, all patients exhibited normal electrolyte or iron levels and no evidence of impaired liver, kidney, heart, or thyroid gland function. Iron deficiency anemia or hypothyroidism have been discussed in the management of PCC before; however, our findings re-iterate that PCC may be a distinct disorder as there were no alternative diagnoses underlying the phenotypes in our patients.

In a recent study, microclotting was reported in adults with PCC, accompanied by elevated levels of key biomarkers associated with endothelial and clotting abnormalities, potentially including thrombotic endothelialitis as a significant pathological process in PCC [32]. Despite such concerns regarding coagulopathies in PCC, coagulation studies were within normal range including D-dimer levels. Since microclots in PCC exhibit exceptional resistance to clot breakdown there might be a lack of notable D-dimer elevation. We discuss that standard coagulation tests may rather serve to exclude alternative diagnoses than aid in establishing a definitive diagnosis of PCC. 

C-reactive protein and erythrocyte sedimentation rate were not elevated in patients with PCC, indicating the absence of systemic inflammation. Currently, there are no other studies available for comparison regarding common laboratory values in children with PCC. There is a lack of a sensitive diagnostic test for or against establishing a PCC diagnosis, and recent publications on adrenergic or muscarinergic autoantibodies have led to several treatment trials with inconsistent results [33], ultimately showing no relevant consequence as biomarkers to our current knowledge.

There are only very few studies yet that comprehensively report laboratory investigations in children and adolescents with PCC. Our primary aim was to identify whether PCC is a distinct disorder without any abnormalities in standard laboratory investigations of liver, kidney, heart, or heart function. Indeed, we did not find any such abnormalities in our cohort through either deep phenotyping on follow-up investigations or molecular investigations. We discuss that such comprehensive investigations are necessary at initial presentation to exclude differential diagnoses, but suggest that follow-up investigations may rather focus on investigating laboratory parameters as evaluated necessary by the treating physician based on manifestation of novel phenotypes or change in existing phenotypes.

### 4.5. Potential Immune Dysregulation as a Risk Factor for Pediatric PCC

As our inclusion criteria implicated a clear link to a COVID-19 infection, we relied on SARS-CoV-2 Spike RBD IgG that is considered the most relevant antibody in this regard [34]. There was significant variability in SARS-CoV-2 Spike RBD IgG antibody levels, which is expected as antibody levels can vary widely between individuals and over time due to their fast decay [34,35,36]. For example, younger children were reported with higher levels than older individuals [35,36]. Whether SARS-CoV-2 Spike RBD IgG antibodies play a specific role in PCC, beyond indicating previous SARS-CoV-2 infection, remains elusive. As serum antibodies decrease over time [37], we discuss that a closer analysis of antibody levels in comparison to PCC manifestation or severity may be warranted in future studies, as it remains elusive whether antibody levels correlate with duration from acute illness until clinical presentation. Recent papers have indeed discussed antibody kinetics in immune response from six months until 616 days in a longitudinal cohort of Spanish primary health care workers [38,39].

It is unclear how acute COVID-19 infections turn into PCC disease courses. A study found increased CD8 T cells in convalescent adults six months after infection and a lower T cell immune response against the SARS-CoV-2 S protein in patients with PCC [20]. This study suggested the persistence of SARS-CoV-2 in mucosal tissues, as indicated by significantly higher specific IgA levels to N and S proteins in PCC patients. To date, no studies on T cell immunity in children with PCC are available for reference. We did not observe any deficiencies or elevations in immunoglobulin levels, except for significantly elevated IgE in nearly half of the patients, indicating an atopic phenotype. Correspondingly, sensitizations to aeroallergens were present in over 90% of the patients, with a particular emphasis on dogs. This marks a massive increase in the rate of sensitizations to aeroallergens compared to the average prevalence in German adolescents aged 14–17 years, which stands at 48% [40]. We discuss that atopic disease should be considered a risk factor for the manifestation of PCC even in the absence of self-reported manifestation of allergic diseases. Atopy is defined as the genetic tendency to develop allergic diseases such as allergic rhinitis, asthma, and atopic dermatitis. As such, young individuals with atopy may not have suffered from noticeable symptoms of allergic diseases at the disease onset of PCC. We discuss that previously published data may have linked allergic disorders with PCC, as a study involving more than 500 children and adolescents had previously demonstrated a higher risk of PCC in children with allergic diseases [41]. As we focus on a cohort with childhood onset PCC, we discuss the genetic susceptibility towards immune dysregulation as a potential risk factor for the manifestation of PCC. Recent reports have discussed pathogenic genetic variants in transcription factors and innate immune response genes in patients developing PCC [42]. Allergies and obstructive lung disease were also identified in larger cohorts of adult patients with PCC [43]. Furthermore, a study conducted in Germany identified allergic rhinitis as an additional risk factor [7]. The pathomechanism of atopic disease in nascent PCC remains elusive. Since immune responses in patients with atopic diseases commonly skew towards a type 2 inflammation, we discuss how general IgE elevation may contribute to a potential immune dysregulation in PCC and propose that screening for IgE levels may give a clearer indication of atopy in the context of PCC.

Analyses of peripheral immune cells including lymphocyte subsets did not reveal any abnormalities. A recent study in adult probands found reduced numbers of CD4 and CD8 effector memory T cells in PCC patients, which were not examined in our patients [44]. However, we did identify significantly elevated IgG3 and IgG4 subclasses in patients with concomitant ME/CFS as a sign for immune dysregulation in particular severe disease courses of PCC. The four human immunoglobulin G subclasses have distinct effector properties due to differences in binding Fc receptors on the surface of immune cells and activating complement. IgG3 is involved in recognizing antigens on viral infections and has been identified in SARS-CoV-2 neutralization in the context of acute COVID-19 illness [45]. IgG4 was identified in IgE-mediated hypersensitivity during the sensitization phase characterized by differentiation and clonal expansion of CD4+ cells producing Th2 cytokines such as IL-4 and IL-13, eventually supporting an isotype switch of B lymphocytes to produce allergen-specific IgE for binding on basophils and mast cells [46]. Recent reports have implicated IL-13 upregulations after acute COVID-19 infections [47]. As IL-13 is involved in IgE production in allergic asthma patients in atopic diseases [48], we discuss that atopic patients with likely elevated IgE may suffer from an aggravation in post-viral immune dysregulation that in turn leads to persistent phenotypes.

While recent reports have found unexpectedly high virus-specific IgG4 levels after more than two mRNA vaccinations [49,50], another report has observed low total IgG4 levels in 64 PCC samples with small but significant difference for the spike protein receptor-binding domain that may contribute to chronic inflammation [51]. As the authors of those recent publications have not clarified the disease severity in the reported patients, our novel finding of significantly upregulated IgG4 levels in patients with deep phenotyping of particularly severe disease course with concomitant ME/CFS may serve as groundwork for future functional studies.

As the reported PCC patients had a higher proportion of atopic patients when compared to the control group, we discuss how these findings may suggest a trial of antihistamines in treating concomitant atopic diseases in PCC patients. However, it is unclear whether any treatment with antihistamines may benefit non-atopic PCC patients and may warrant future research on this topic.

### 4.6. Recovery

More than half of the patients in this study experienced recovery from PCC, and among those with ongoing symptoms, the majority reported substantial improvement. These findings are in line with the current literature, as PCC is generally considered a self-limited and reversible disorder [22,23]. However, in a subset of children, symptoms may persist for longer than 12 months. In many cases, patients reported a gradual improvement, with symptom-free intervals becoming increasingly frequent until no complaints remained. For those who experienced ME/CFS, pacing and interval-based regaining of motor activity played a crucial role in their recovery, so as to limit the frequency and severity of relapses while remaining as active as possible.

### 4.7. Strengths and Limitations

We acknowledge that the small sample size in our monocentric study is an important limitation. There is a lack of literature on real-world data from deep phenotyping in pediatric PCC as a rare disease. However, unlike many studies with large sample sizes that rely solely on questionnaires for quantification of patient phenotypes, our study has focused on qualitative interviews during patient recruitment that included thorough examinations by at least one pediatric physician specialized on PCC and relevant consultants as required to provide groundwork for future studies on this topic. The careful deliberation included comprehensive laboratory diagnostics and pulmonary function tests were conducted for every patient that may indeed prove challenging in a pediatric setting. As a result, the dataset presented here is comprehensive and built on high data reliability.

Given that patients presenting with suspicion of PCC often have a long history of symptoms and significantly impaired quality of life, we discuss that deep phenotyping is crucial but also challenging and time-consuming during out-patient clinics. If patient history suggests PCC, further diagnostic evaluation screens for associated conditions should include comprehensive blood laboratory investigations, a pulmonary function test in case of dyspnea, and a standardized neurological examination in case of Myalgic Encephalomyelitis/Chronic Fatigue Syndrome (ME/CFS) or depression via questionnaires and psychological evaluation. Further investigations may include lumbar punctures, brain magnetic resonance imaging (MRI), or skin punch biopsies for small fiber neuropathy, depending on the phenotype. Diagnostic investigations are generally limited in children due to pain management and potential need for anesthesia. We discuss that PCC remains a diagnosis of exclusion for which pediatric specialists carefully consider the diagnostic approach based on the patient history and discuss an adequate management with the patient’s family. Further studies are needed to gain insight into the underlying pathophysiology of pediatric PCC.

## 5. Conclusions

In conclusion, our findings in the diagnosis and management of PCC in children and adolescents present a high prevalence of sensitizations to aeroallergens, observed in over 90% of patients, suggesting that presence of atopic disorders may be a risk factor for PCC regardless of reported manifestation of allergic symptoms. We propose to screen patients with PCC for elevation of IgE levels in future studies. Moreover, in patients with concurrent ME/CFS, we identified notably elevated levels of IgG3 and IgG4, suggesting immune dysregulation as a potential risk factor for severe disease courses. In severe cases, regular school attendance was often disrupted for long periods of time, marking a significant break into the social and mental well-being of children and adolescents at a particularly formative period of life. Nonetheless, the majority of patients experienced complete recovery, and even those who had not fully recovered showed significant improvement in their symptoms. This report of a monocentric investigation of patients with PCC with deep phenotyping, molecular investigations, and follow-up on disease courses adds further insight into management and counseling of patients and families. 

## Figures and Tables

**Table 1:** Molecular investigations for organ dysfunction; mean values and standard deviations (SD) from 21 children and adolescents with PCC.

Category	Parameter	Unit	Mean	SD
Electrolytes	Sodium	mmol/L	138.7	1.77
	Potassium	mmol/L	3.87	0.33
	Chloride	mmol/L	103.0	9.5
	Calcium	mmol/L	2.39	0.097
	Phosphate	mmol/L	1.35	0.23
Liver Function	Alanine aminotransferase	U/L	24.14	5.2
	Aspartate aminotransferase	U/L	18.67	7.66
	γ-Glutamyltransferase	U/L	17.57	8.93
	Glutamate dehydrogenase	U/L	1.37	1.13
	Cholinesterase	kU/L	8.08	2.21
	Bilirubin	mg/dL	0.41	0.36
Skeletal and Cardiac Muscle	Creatine kinase	U/L	107.1	47.58
	Creatine kinase-MB	U/L	14.95	4.55
	Troponin T-high sensitivity	µg/L	0.0019	0.0044
	NT-proBNP	ng/L	47.6	45.84
Kidney Function	Total protein	g/L	74.33	4.61
	Albumin	g/L	47.43	3.3
	Creatinine	mg/dL	0.63	0.17
	Blood urea nitrogen	mg/dL	26.14	5.28
	Uric acid	mg/dL	4.46	0.86
Thyroid Function	Thyroid-stimulating hormone	mU/L	2.173	1.06
	Free thyroxine (T4)	ng/L	12.42	1.64
Coagulation	International normalized ratio		1.043	0.10
	Activated partial thromboplastin time	s	26.1	2.61
	Fibrinogen	g/L	2.59	0.45
	D-dimer	mg/L	0.23	0.27
Others	Blood sugar	mg/dL	86.8	9.5
	Lactate dehydrogenase	U/L	203	34.34
	Iron	µmoL/L	14.58	7.12
	Ferritin	µg/L	40.95	18.63
	Transferrin	g/L	2.8	0.5
	Transferrin saturation	%	21.62	11.74
	Lipase	U/L	27.89	19.53
Inflammation	C-reactive Protein	mg/L	0.62	0.9
	Erythrocyte sedimentation rate	mm/h	4.47	2.39

**Table 2:** Immunological diagnostics; mean values and standard deviations (SD) from 21 children and adolescents with PCC. Elevated levels are marked in bold.

Category	Parameter	Unit	Mean	SD
Immunoglobulins	IgG	g/L	10.66	2.86
	IgG1	mg/L	6014	2015
	IgG2	mg/L	2865	1530
	IgG3	mg/L	863	1130
	IgG4	mg/L	538	453
	IgA	g/L	1.24	0.64
	IgM	g/L	1.11	0.57
	**IgE**	**kU/L**	**174.2**	**204.3**
	SARS-CoV-2 Spike RBD IgG	BAU/mL	1233	1707
Complete Blood Count	Leukocytes	10^9^/L	6.85	2.11
	Erythrocytes	10^12^/L	4.92	0.44
	Hemoglobin	g/dL	13.72	1.07
	Hematocrit	%	40.48	3.03
	Mean corpuscular volume	fl	82.57	2.48
	Mean corpuscular hemoglobin	pg	28.05	1.28
	Mean corpuscular hemoglobin concentration	g/dL	82.57	2.48
	Red cell distribution width	%	12.49	0.58
	Mean thrombocyte volume	fl	9.7	0.95
	Normoblasts, rel.; abs.	%; 10^9^/L	0; 0	0; 0
	Neutrophils, rel.; abs.	%; 10^9^/L	49.27; 3.35	11.2; 1.36
	Immature granulocytes, rel.; abs.	%; 10^9^/L	0.26; 0.017	0.08; 0.011
	Lymphocytes, rel.; abs.	%; 10^9^/L	39.82; 2.76	11.04; 1.40
	Monocytes, rel.; abs.	%; 10^9^/L	7.39; 0.48	2.49; 0.17
	Eosinophils, rel.; abs.	%; 10^9^/L	2.31; 0.16	1.35; 0.12
	Basophils, rel.; abs.	%; 10^9^/L	0.60; 0.043	0.26; 0.027
Lymphocyte Subsets (FACS)	Lymphocytes, rel.; abs.	%; 10^9^/L	39.26; 2.7	8.81; 1.16
	T lymphocytes CD3+, rel.; abs.	%; 10^9^/L	71.76; 1.9	7.71; 0.73
	T lymphocytes CD3+HLA-DR+, rel.; abs.	%; 10^9^/L	8.12; 0.14	6.8; 0.12
	T lymphocytes CD4+, rel.; abs.	%; 10^9^/L	42.83; 1.12	8.14; 0.46
	T lymphocytes CD8+, rel.; abs.	%; 10^9^/L	23.40; 0.63	4.67; 0.28
	T lymphocytes CD8+CD38+, rel.; abs.	%; 10^9^/L	66.06; 0.37	10.53; 0.16
	CD4/CD8 ratio		1.98	0.7
	B lymphocytes CD19+, rel.; abs.	%; 10^9^/L	16.85; 0.49	5.76; 0.37
	NK cells CD16+CD56+, rel.; abs.	%; 10^9^/L	9.87; 0.27	5.1; 0.21

Abbreviations: RBD: receptor-binding domain; rel.: relative; abs.: absolute; CD: cluster of differentiation; FACS: Fluorescence activated cell sorting; NK: natural killer.

## Data Availability

The data presented in this study are available on request from the corresponding author. The data are not publicly available due to privacy restrictions.

## Appendix A

**Table A1:** Criteria for Myalgic Encephalomyelitis/Chronic Fatigue Syndrome (ME/CFS), modified from International Consensus Criteria with additional criteria for gastrointestinal disorders [17]. Diagnosis with ME/CFS required all criteria in A (exhaustion after exertion), at least one criterion in three out of four sub-categories of B (neurological disorders), and at least one criterion in each C (immunological and autonomic disorders) and D (cardiorespiratory and temperature stability disorders).

Category	Sub-Category	Criteria
A. Exhaustion after exertion		Significant new and persistent physical/mental exhaustion
		Increased general feeling of sickness and/or pain after exertion
		Significant exhaustion after exertion
		Significantly delayed recovery periods (hours to days)
		Low tolerance to physical/mental exertion, reduced activity level
B. Neurological disorders	B.1 Neurocognitive impairments	Difficulties in processing information (concentration, perceptual problems, confusion)
		Impairment of short-term memory (word-finding, reading disorders, impaired working memory)
	B.2. Pain	Significant muscle and/or joint pain
		Headaches (including migraines, ascending neck pain)
	B.3. Sleep disturbances	Difficulty falling asleep/staying asleep, altered day-night rhythm
		Unrestful sleep, daytime sleepiness
	B.4 Neurosensory, perceptual or motor disorders	Sensory/perceptual disorders (difficulty seeing, hearing, smelling, tasting, touching)
		Movement disorders (incoordination, muscle weakness, gait disturbance)
		Sensory/perceptual disorders (difficulty seeing, hearing, smelling, tasting, touching)
		Movement disorders (incoordination, muscle weakness, gait disturbance)
C. Immunological or autonomic disorders		Flu-like symptoms (painful lymph nodes, sore throat, feeling sick)
		Susceptibility to viral infections with prolonged recovery periods
		Gastrointestinal disorders (nausea, diffuse pain, burning sensation, bloating)
		Bladder dysfunction
		New onset of intolerance to foods, medications, odors
D. Cardiorespiratory or temperature stability disorders		Cardiovascular (dizziness, palpitations and/or “blacking out” with changes in position; palpitations)
		Respiratory (shortness of breath on light exertion, difficulty breathing, fatigue of auxiliary respiratory muscles)
		Loss of thermostatic ability (body temperature adjustment disorder, sweating, feverish feeling, cold extremities)
		Lack of tolerance for extreme temperatures
